# Sleep Impairment and Psychological Distress among Patients with Inflammatory Bowel Disease—beyond the Obvious

**DOI:** 10.3390/jcm9072304

**Published:** 2020-07-20

**Authors:** Georgiana-Emmanuela Gîlcă-Blanariu, Gabriela Ștefănescu, Anca Victorița Trifan, Mihaela Moscalu, Mihail-Gabriel Dimofte, Cristinel Ștefănescu, Vasile Liviu Drug, Vlad-Adrian Afrăsânie, Manuela Ciocoiu

**Affiliations:** 1 Faculty of Medicine, Grigore T. Popa University of Medicine and Pharmacy, 700115 Iasi, Romania; georgiana.gilca@gmail.com (G.-E.G.-B.); ancatrifan@yahoo.com (A.V.T.); gdimofte@yahoo.com (M.-G.D.); cristinel.stefanescu@gmail.com (C.Ș.); vasidrug@email.com (V.L.D.); vlad_afrasanie@yahoo.com (V.-A.A.); mciocoiu2003@yahoo.com (M.C.); 2 Institute of Gastroenterology and Hepatology, Sf Spiridon County Clinical Emergency Hospital, 700111 Iasi, Romania; 3 Second Department of Oncologic Surgery, Regional Institute of Oncology, 700483 Iasi, Romania; 4 Fifth Department of Psychiatry, Socola Institute of Psychiatry, 700282 Iasi, Romania

**Keywords:** inflammatory bowel disease, sleep impairment, psychological distress, PSQI, HADS

## Abstract

Background: A healthy sleep–wake cycle is fundamental for regulating immune function. Sleepiness and fatigue are often manifestations of chronic inflammatory disorders, such as inflammatory bowel disease (IBD), potentially influencing the course of the disease. Our aim was to characterize sleep impairment in patients with IBD and to identify potential associated factors. Methods: We conducted a single-center prospective case control study including IBD patients and healthy controls. We evaluated clinical and biochemical parameters, sleep impairment through Pittsburgh Sleep Quality Index (PSQI) and anxiety and depression through Hospital Anxiety and Depression Scale (HADS) questionnaires. Results: In total, 110 patients with IBD and 66 healthy controls were included. Patients with IBD had a significantly altered sleep quality compared to the control group (*p* < 0.001), with sleep impairment also occurring for patients in remission (median PSQI = 7), but without significant differences between ulcerative colitis and Crohn’s disease. However, PSQI was correlated with disease activity scores only for ulcerative colitis and not for Crohn’s disease. Among patients with increased PSQI, only 30.19% used sleep medication. Sleep impairment was significantly correlated with altered psychological status (*p* < 0.01) and the presence of extraintestinal manifestations (*p* = 0.0172). Conclusions: Sleep impairment is frequent among patients with IBD, is associated with psychological distress and several disease-related parameters and should be routinely evaluated, at least in several IBD patient subgroups, to improve disease management.

## 1. Introduction

The main types of inflammatory bowel disease (IBD), ulcerative colitis (UC) and Crohn’s disease (CD) are chronic relapsing inflammatory conditions with a complex and incompletely understood pathogenesis. Part of this intricate pathogenesis is attributed to the interaction between genetics and environmental factors [1]. Since the genetic background is complex and difficult to target, increasing attention has been attributed to modifiable environmental factors.

As an environmental factor, sleep seems to have important influence on the cellular immunity and has also been linked to increased levels of various proinflammatory mediators such as interleukin-1 and tumor necrosis factor-α [2], which influence IBD activity. Decreased sleep duration has been associated with an increased risk of the development of UC [3] and sleep impairment has been commonly associated with a reduced quality of life among patients with IBD [4]. Impaired sleep has been associated with disease activity in IBD patients [5,6], but altered sleep quality has been reported both for active and quiescent disease [5,7]. 

Considering that medical and psychological stability are connected within the complex pathophysiology of IBD, and that certain symptoms seem to impact, as well as be affected by, both physical and psychological factors [7], studying the interrelationship between sleep, psychological distress and disease characteristics appears to be a promising method and targeting sleep improvement might represent a modifiable determinant of IBD related outcomes. The present research aimed to investigate the severity of sleep impairment in patients with IBD vs. a healthy control group and to identify the potential relationship between poor subjective sleep, IBD activity and characteristics and psychological distress.

## 2. Materials and Methods

This study was a single-center case control study conducted at the Institute of Gastroenterology and Hepatology, Sf Spiridon County Clinical Emergency Hospital, Iași, Romania, and the Regional Oncology Institute, Iasi, Romania, investigating the hypothesis that, among adult IBD patients, there is an impaired quality of sleep compared to normal subjects and aiming to identify particular aspects of sleep impairment in this patient category. Consequently, we first calculated the sample size and established the inclusion and exclusion criteria; herein, we report all measured data.

### 2.1. Sample Size Calculation

Taking into account that there is a significant variation for reported sleep impairment among IBD patients, varying between 73 and 100% among patients with active disease and between 48 and 72% in patients with inactive disease [5,8] and also that there is no data regarding sleep impairment in Romanian patients with IBD, we considered an effect size of 92.5% to represent the frequency of IBD cases exhibiting impaired sleep. Establishing the optimum sample size involves obtaining a minimum volume to ensure an appropriate representativeness of the patient category. In order to achieve this prerequisite, we established a confidence interval of 95%. The sample size was determined based on the condition of having a lower confidence interval than the allowed error level. Consequently, we used the equation:

(1) $$n\geq {\left(Z_{\left(1-\frac{\alpha }{2}\right)}\right)}^{2}\cdot \frac{p\cdot \left(1-p\right)}{d^{2}},$$

with *Z* = 1.96 for a 95% confidence interval, and a *d* value corresponding to a 5% estimation error. We estimated the sample size as having a higher value, based on an effect size of 92.5%. For a maximum assumed error of 5%, the minimum sample size should reach 106 cases.

### 2.2. Subject Recruitment

For the study group, we prospectively and consecutively recruited patients with IBD who were evaluated in our department during a 6-month period, between September 2019 and February 2020. Inclusion criteria were age above 18, written informed consent, established diagnosis of IBD based on clinical, endoscopic and histological findings, following the current guidelines. The exclusion criteria included refusal to participate in the study and various aspects which might influence sleep quality [9]: pregnancy, history of neoplasia, previously diagnosed psychiatric disorders, history of alcohol or drug use, acute infections, sleep apnea, BMI > 35, significant major associated illness (cardiopulmonary disorders, cirrhosis, or significant chronic renal impairment or active malignancy), night shift workers, and patients taking medications (analgesics, muscle relaxants, antineoplastic agents, phenytoin, amphetamines, prescription weight-loss drugs, thyroid medication, and anticholinergic/antihistamine medications).

### 2.3. Evaluated Parameters

The clinical and anamnestic data were collected during patient evaluations and also by reviewing patients’ charts to assessing their demographic data and supplementary clinical information (extraintestinal manifestations, current and previous medication, IBD-related surgery) To evaluate disease activity, the Mayo score was used for UC and Crohn’s Disease Activity Index (CDAI) for CD assessment, together with biochemical markers of systemic inflammation (C reactive protein (CRP), erythrocyte sedimentation rate (ESR), fibrinogen) and fecal calprotectin measurement; CRP/albumin and neutrophil-to-lymphocyte (PMN/Ly) ratios were also evaluated. Sleep disorders were assessed using the Pittsburgh Sleep Quality Index (PSQI) and psychological status was assessed using the Hospital Anxiety and Depression Scale (HADS).

In order to characterize sleep disturbance patterns specific to patients with IBD, we selected a control group, consisting of healthy volunteers, who were recruited among patients with no digestive complaints and with negative colonoscopy findings at screening colonoscopy. Exclusion criteria were similar to the ones in the study group and involved factors which have an impact on sleep quality. Clinical and demographic data were also collected for the control group. All subjects enrolled in the control group were asked to respond to the questions included in two questionnaires, PSQI and the HADS, evaluating sleep impairment and psychological distress.

### 2.4. Description of Instruments Used for Assessment of Sleep and Psychological Impairment

PSQI is a widely used sleep evaluation tool, both in research [10] and in clinical practice, which reflects the quality of sleep through seven components and considers recalling events in the past month. This questionnaire includes nineteen individual items, which concur to generate 7 components referring to subjective sleep quality, use of sleeping medication, sleep latency and duration, habitual sleep efficiency, daytime dysfunction. Patients can evaluate each component and the result can range from “0” in cases of no impairment to “3” in cases of severe difficulty. Consequently, the sum of ratings from each component lead to scores ranging from 0 to 21. Obtaining a total score of 5 or above corresponds to an impaired quality of sleep, with a diagnostic sensitivity of 89.6% and a specificity of 86.5% [5,11,12].

Anxiety and depression were evaluated using HADS, which includes 7 items, with scores ranging from 0 to 3, leading to total scores from 0 to 21. The higher the score, the more severe the symptoms, with a threshold of 11 being considered clinically significant for the diagnosis of anxiety/depression, while scores ranging from 8 to 10 suggest a mild disorder [13]. In the performed study, we considered scores ≥8 as compatible with anxiety or depressive moods.

Both questionnaires were administered by the same interviewer, since they have not been validated in the Romanian language for use in IBD patients and to minimize different interpretations or misunderstandings regarding the questions.

### 2.5. Statistical Analysis

The statistical analysis of data was performed using SPSS version 24 (IBM Corporation, North Castle Drive, Armonk, NY, USA). Continuous variables were reported as mean values and standard deviation, or as median with 25th–75th percentiles. The comparisons between the analyzed groups were performed using Student’s t-test, the Mann–Whitney U Test or the Kruskal–Wallis test for continuous variables, depending on the homogeneity of the data series, based on Levene’s test. For comparison among more than two groups, appropriate post-hoc tests were applied. The qualitative variables were presented as absolute (*n*) and relative (%) frequencies and the comparison among the groups was based on the results of McNemar, Yates or Pearson chi-square tests. The univariate correlation analysis was completed based on the results of Spearman Rank Order Correlations tests. The multivariate analysis of prognostic factors for PSQI values was achieved using an ordinal regression model. The prediction power of some variables was assessed, based on the ROC curve, by evaluating the area under the ROC curve (AUC). The calculated significance level (*p*-value) within the applied tests was considered significant for *p* < 0.05.

### 2.6. Ethical Considerations

The study protocol and all included procedures performed in the study were in accordance with the ethical standards of the institutions’ Ethical Review Boards and with the 1964 Helsinki declaration and its later amendments or comparable ethical standards. Ethical approval was obtained from Grigore T Popa University of Medicine and Pharmacy, Iași, (25.Nov.2018) and from all institutions involved where patients and controls were recruited, namely Sf Spiridon County Clinical Emergency Hospital (No 45/04.09/2019) and the Regional Oncology Institute (No. 202/21.06.2018). Informed consent was obtained from all individual participants included in the study.

## 3. Results

### 3.1. Descriptive Statistics

In the study, we included 110 patients, 54 female and 56 male patients, with a mean age of 45.1 years, with no statistical differences between the study group and the control group regarding demographic data and 66 healthy controls (Table 1).

Regarding the biochemical parameters, which were evaluated only in the study group, we identified significant differences between UC and CD for hemoglobin level and the presence of extraintestinal manifestations (EIM); all the other evaluated parameters did not differ significantly between UC and CD. Both UC and CD subgroups included patients in remission and with active disease (UC—57.89% for active disease and CD—29.41% for active disease), based on Mayo and CDAI scores, highlighting a significant difference concerning disease activity between the disease subtype (*p* = 0.0201), with a higher percentage of patients with active disease among the UC subgroup (Table 2).

### 3.2. Sleep Quality among Studied Groups

**Figure 1:**
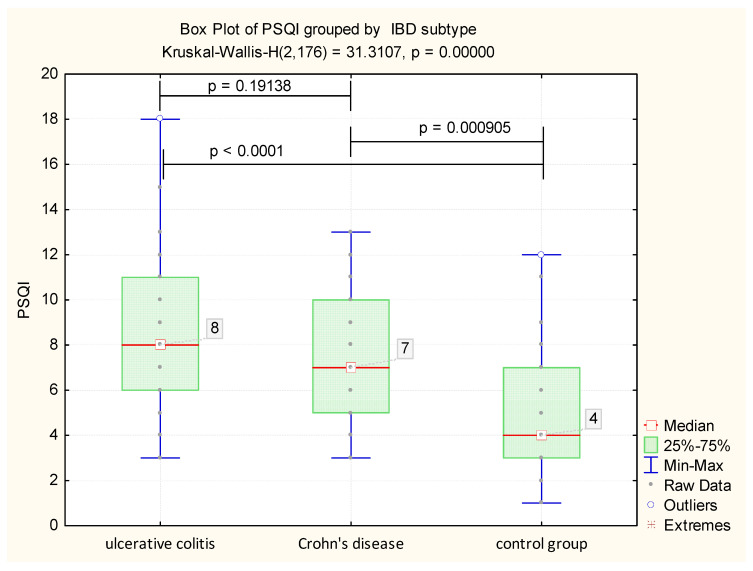
Evaluation of global Pittsburgh Sleep Quality Index (PSQI) scores among the studied groups.

Sleep quality was impaired in patients with IBD compared to the control group (H = 31.3107, *p* < 0.001), with a median PSQI of eight in IBD patients, compared with the control group, where the median PSQI was four. When evaluating PSQI among IBD subtype, there was no statistically significant difference in sleep quality between CD and UC patients (*p* = 0.1913) (Figure 1).

Not only did we find higher statistically significant values for PSQI scores of eight in the study group (5; 10) versus four (3; 7) in the control group (*p* < 0.0001), we also identified significantly higher values for HADS-Anxiety (HADS-A) and HADS-Depression (HADS-D) among IBD patients compared to the control group, with a median (Q25;Q75) for HADS-A of seven (5; 10), and a median (Q25; Q75) for HADS-D of eight (5; 11) for patients with IBD (Table 3).

### 3.3. Influence of Disease Activity on Quality of Sleep

**Figure 2:**
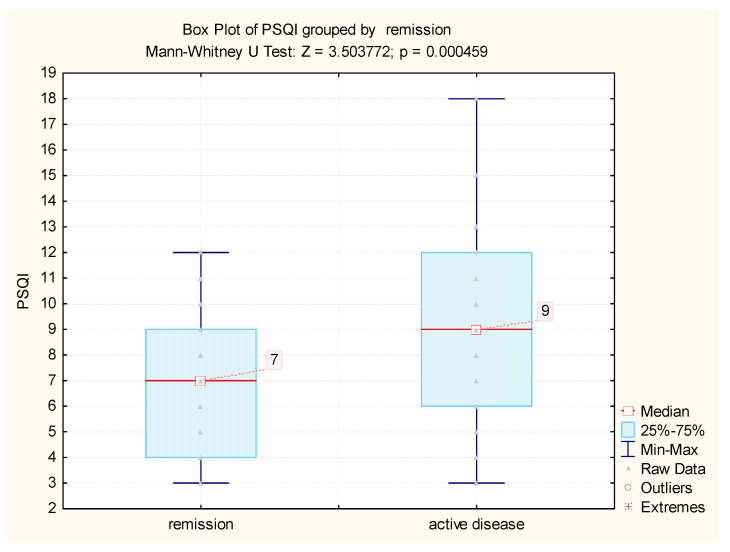
The differences in PSQI value between remission and disease activity.

Disease activity played a significant role in sleep quality, since patients experiencing a disease flare-up had statistically significant higher PSQI scores than patients in remission. Although there was a statistically significant difference in PSQI between patients with active disease, compared to remission, when analyzing the subgroup of patients in remission, the values of PSQI were also increased, with a median value of seven (while in the control group the median was at the upper value of the normal range—a score of four (Figure 2).

In order to further characterize the influence of disease activity on sleep impairment, the correlation between disease activity through Mayo and CDAI scores was also studied, reflecting a significant correlation of PSQI values with Mayo score (*p* < 0.0001), but not with CDAI score (*p* = 0.982) (Table 4).

### 3.4. Exploring the Link between Sleep Impairment and Various Patient-Related Parameters

Apart from disease activity, we have also evaluated whether several patient characteristics (such as age and the presence of extraintestinal manifestations) and biochemical markers (hemoglobin, CRP, fibrinogen, fecal calprotectin, albumin) are correlated with PSQI score. While age and the presence of anemia (reflected through the level of hemoglobin) were not significantly correlated with sleep disorders, the presence of extraintestinal manifestations was significantly correlated with PSQI score, at a level of statistical significance (Table 5). There was a statistically significant correlation between markers of inflammation (CRP, fibrinogen and calprotectin) and PSQI score among IBD patient group. Moreover, PMN/Ly and CRP/albumin ratios were also significantly correlated with PSQI score, when applying Spearman Rank order correlation (Table 5).

### 3.5. Identifying Cut-Off Values of Various Parameters as Predictors of Impaired Sleep

**Figure 3:**
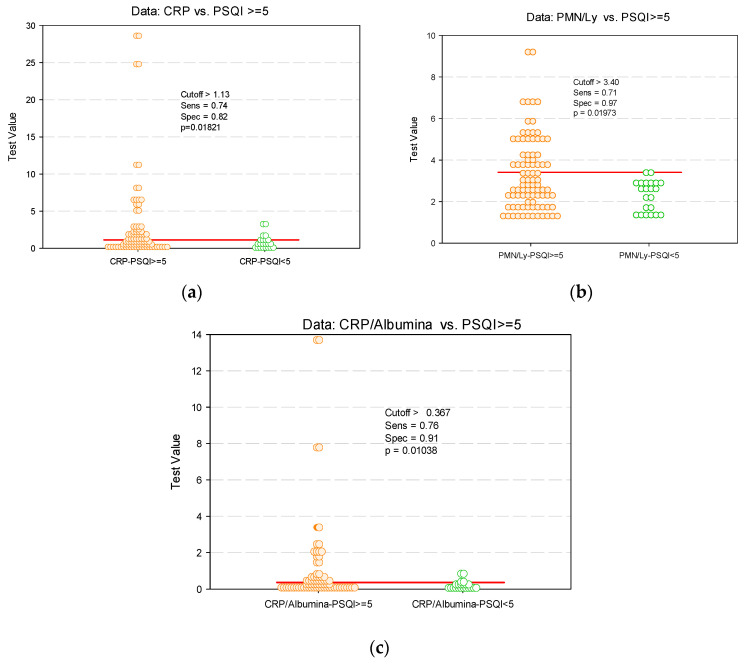
Identifying cut-off values predictive for sleep impairment (dot histogram) for: (**a**) CRP; (**b**) PMN/Ly ratio; (**c**) CRP/albumin ratio.

Considering the correlation between CRP, PMN/Ly ratio and CRP/albumin ratio with PSQI scores, we used ROC curve analysis to obtain the cut-off for these ratios, which could predict an impaired sleep quality reflected in PSQI scores above five. We identified a cut off value of 3.4 (AUC = 0.6612; 95% CI: 0.5519–0.7705, *p* = 0.0197), with 71% sensibility and 97% specificity for PMN/Ly ratio as predictor of impaired sleep (PSQI ≥ 5). Regarding the value of CRP/albumin ratio, we identified a cut-off value of 0.367 (AUC = 0.6806, 95%CI: 0.5576–0.8036; *p* = 0.01038), with a sensibility of 71% and specificity of 91% as a predictor of impaired sleep (Figure 3).

**Figure 4:**
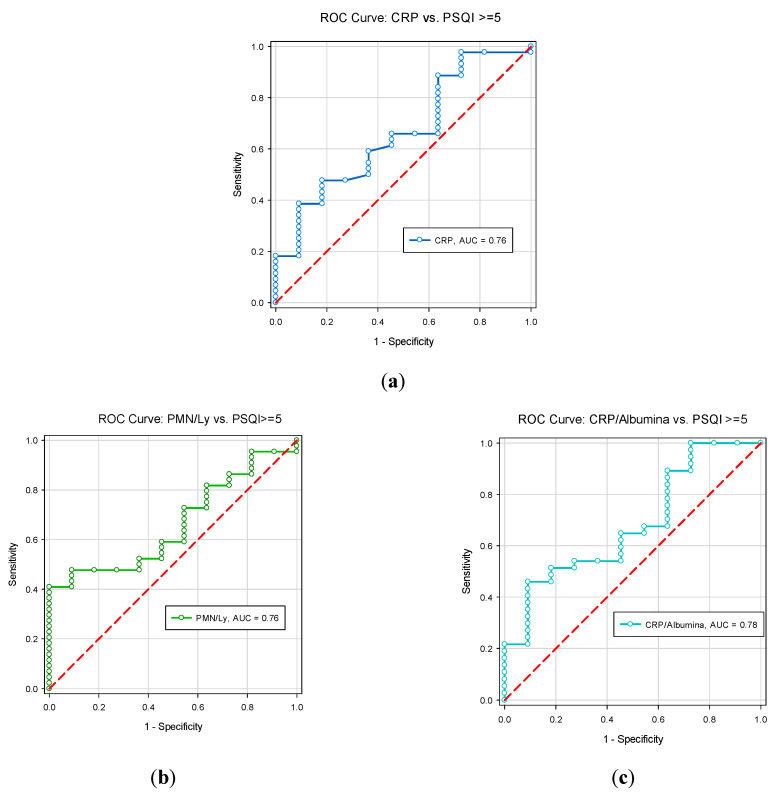
ROC curves evaluating the prediction power of (**a**) CRP, (**b**) PMN/Ly ratio, (**c**) CRP/albumin ratio on PSQI values.

The comparative analysis of AUC values for CRP, PMN/Ly ratio and CPR/albumin ratio regarding the prediction of PSQI values demonstrated comparable predictability for PSQI values among the analyzed situations, with a slightly higher value for CRP/albumin ratio (Figure 4).

### 3.6. Exploring the Link between Sleep Impairment and Psychological Distress

When evaluating the influence of psychological distress on sleep impairment, there was a strong correlation between PSQI and the values of HADS, both for anxiety and depression in the IBD group. (Table 6).

Furthermore, no patients receiving sleep medication had a normal PSQI and, among patients with an increased PSQI, there was an important percentage of patients with IBD (69.81%) receiving no therapy for sleep impairment.

### 3.7. Highlighting Particular Aspects Related to PSQI Components

When further analyzing the PSQI subscores and the rankings (from zero—no alteration to three—major alteration) attributed to each component, all main components of the score contributed to poor sleep quality in patients with IBD compared to the control group, except for Component 6—use of sleep medication, which did not differ between the study group and the control group (Table 7).

Taking into account the statistically significant differences between patients with IBD and the control group regarding most of the components of the PSQI score, we conducted a multivariate analysis in order to rank the contribution of each component to the total score. This analysis highlighted that, in both IBD and the control group, the highest contributor to the score was the subjective evaluation of sleep quality (Component 1); however, all further components ranked differently between the two groups (Table 8 and Table 9).

### 3.8. Influence of Therapy on Sleep Quality

**Figure 5:**
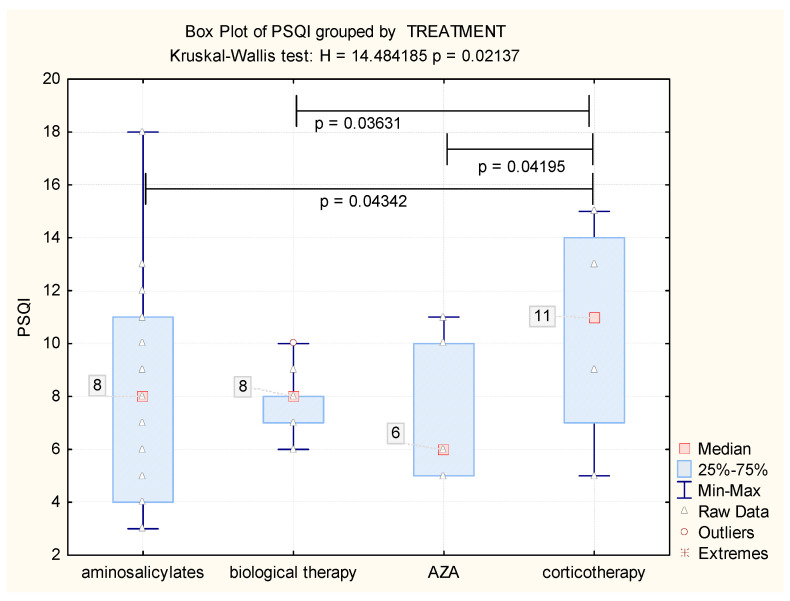
The values of PSQI score among various treatment options.

To study the factors involved in sleep impairment within IBD more closely, we evaluated the role of therapy in this respect. We identified higher statistically significant values of PSQI in the subgroup of patients undergoing corticotherapy (*p* < 0.05), with a median score of 11, compared to all other subgroups. Among the PSQI scores corresponding to the other subgroups (aminosalycilates, azathioprine, biological therapy), there were no statistically significant differences. In these subgroups, the median of the PSQI was eight (3; 18)—for patients under biological therapy and aminosalicylates—and six (5; 10) for patients under azathioprine treatment (Figure 5).

## 4. Discussion

To our knowledge, this is the first study investigating sleep disturbances among patients with IBD in Romania. Sleep quality in the studied group was significantly impaired compared to the control group, a finding that has been confirmed by various other recent studies [14,15,16].

We highlighted statistically significant differences in quality of sleep within the IBD group, related to the disease activity of IBD, based on the scores used in clinical practice (Mayo and CDAI scores) (active disease: nine (6; 12); remission: seven (4; 9)). Noteworthily, even under these circumstances, the median value of PSQI was seven, within the pathological range, for IBD patients in remission. When analyzing the correlation between disease activity and sleep impairment, there was a statistically significant correlation between PSQI and Mayo score, but not with CDAI score, which might reflect that the relation between the sleep pattern and disease activity could be different between UC and CD, aspect which is supported by other studies, which have highlighted that defective sleep increases the risk of relapse in patients with inactive CD but not UC [17].

In the pursuit of characterizing sleep impairment among patients with IBD, we did not only find a significant impairment in patients with active disease, but also identified significant correlations between PSQI score and all evaluated inflammatory markers (CRP, fibrinogen, fecal calprotectin). Our results are similar to the ones reported by C. Marinelli and collaborators [15]. However, there is conflicting data in the literature regarding the correlation between PSQI and systemic inflammatory markers, considering that other studies did not report a significant correlation [14]. Furthermore, we identified a significant correlation between sleep impairment and extraintestinal manifestations (EIM), which were represented mainly by joint disease (26.31%—UC group and 5.88%—CD patients). This finding might be explained by the presence of inflammatory joint pain leading to awakenings; however, when analyzing specific PSQI findings, there was no significant difference between awakening due to joint pain between patients with IBD and the control group. Consequently, the association between the presence of EIM and impaired sleep in our IBD group might be attributed to a more severe disease course in these patients. Considering the potential relationship between fatigue and sleep impairment, reflected through daytime dysfunction and taking into account that fatigue could be partly related to anemia, we investigated whether anemia plays a significant role in this context. Therefore, we studied the correlation between low hemoglobin level and impaired sleep, which was not statistically significant, a finding that is similar to other reported data [15].

When analyzing the medication use within the IBD group, we identified a statistically significant impaired sleep quality among patients undergoing corticotherapy, with similar findings reported in other studies [18]. Considering the place of corticosteroids in the therapeutic approach to IBD, namely in inducing remission in moderate–severe disease, it is expected that patients undergoing steroid treatment are more likely to have impaired sleep. However, the reason behind this finding may reside not only on an increased disease activity present in this patient subgroup, but could also be partly related to the side effects of steroid treatment. The link between corticosteroid use and poor sleep might represent one argument for opting for corticosteroid-sparing agents in targeting the control of IBD activity. Moreover, a prospective study conducted by Stevens and collaborators demonstrated that the use of anti-TNF therapy and vedolizumab might even lead to an improvement in sleep, depression, and anxiety in patients with moderate to severe IBD, highlighting this aspect within 6 weeks of therapy, which was maintained up to 1 year [19]. Among the other types of medication in our IBD patient group, there was no statistically significant difference in PSQI scores, which might suggest that this type of medication is either associated with a less severe disease phenotype (aminosalicylates) or with a better control of disease activity and less psychological distress (biological therapy and azathioprine).

A particular aspect identified in the study group was the extremely low use of sleep medication (supplements and psychotropic agents), with it only being used by 13.16% of the ulcerative colitis patients and 17.65% of the Crohn’s disease patients, despite a high prevalence of sleep impairment and psychological distress, and also despite previous reports of high sleep medication use among patients with IBD [6,20]. Furthermore, the use of sleep medication was not significantly different between the study group and the control group, although sleep impairment was significantly higher in the IBD group. This might be related to the low number of IBD patients who present as suffering from sleep impairment and psychological distress to psychiatric support, due to a fear of stigmatization, considering that these are mainly young patients, many already with the burden of having been diagnosed with a chronic disease.

Our study poses some limitations, which should be considered, and which highlight the need for further research in the field. Firstly, we only performed a single-center study in a tertiary center, which might lead to the selection of more severe cases; nevertheless, our study also included patients in remission, showing that impaired sleep is also present in this patient subgroup. Secondly, the use of a self-reported measurement method and retrospective reporting of sleep quality, without an objective quantifiable method, might represent a limitation. Using objective measurements (such as polysomnography, actigraphy and even electroencephalography measures) besides sleep questionnaires to evaluate sleep impairment and related daytime dysfunction could represent a potential future direction for improving the knowledge on sleep patterns among patients with IBD. Regardless, our data strongly suggest that the impairment of sleep is common among IBD patients, both in active disease and during remission. Furthermore, a study investigating sleep disorders in patients with IBD, using both the PSQI questionnaire and polysomnography identified significant correlations between some of the parameters evaluated in the PSQI and the results of the polysomnography, such as reported sleep duration and sleep efficiency [7]. Thirdly, the correlations between disease activity and PSQI were based on activity scores (Mayo and CDAI) and we did not include exclusively objective instruments for assessing disease severity (colonoscopic aspect and histology). However, we did include fecal calprotectin level when evaluating disease activity, which has been listed as a good option to substitute the objective evaluation of disease activity through colonoscopy.

Taking into account the high prevalence of sleep impairment among patients with IBD identified in our study, as well as in other studies [14,15,21], and the significant association between sleep impairment and inflammatory parameters, we noticed a potential clinical benefit in identifying cut-off values for several parameters (PMN/Ly ratio, CRP/albumin ratio) that represent indirect measures of active disease as predictors of impaired sleep in patients experiencing a disease flare-up. In the performed study, we identified the high specificity (>90%) of these parameters (PMN/Ly ratio, CRP/albumin ratio), which was compatible with the increased PSQI values. Consequently, a standard evaluation of sleep impairment could be considered, at least for IBD patients with compatible biochemical inflammatory syndrome, while a solution for screening sleep impairment for patients in remission should also be considered. Further studies are needed in order to evaluate the benefit and usefulness of identifying cut-off values for these ratios among patients with active IBD, to establish whether this can represent a viable solution for investigating sleep disruption as a comorbidity among patients with IBD.

Offering complete care for patients with IBD should not only involve the management of inflammation, but also the management of daily stressors, which highly influence quality of life, but can also impact disease evolution. Considering that an outpatient department schedule might be extremely tight, the routine assessment of sleep impairment using objective instruments may be very difficult and inefficient to implement [2]. One potential way to counterbalance this drawback might be screening patients with IBD who fulfill a certain biochemical profile, as suggested, or to bring sleep evaluations of IBD patients into clinical practice by using questionnaires such as PSQI. As suggested in the literature, this second option could be applied by offering them to patients to be completed in the waiting room or to be previously sent and filled in through electronic mail [2]. By using such approaches, healthcare providers managing patients with IBD could both improve patient assessment and help us to move closer to a more integrative strategy.

## 5. Conclusions

Our study highlights the high impact of sleep impairment on patients with IBD, emphasizing the opportunity of including sleep quality assessment in the routine evaluation of this patient category. However, the optimal method to therapeutically address this issue—changes in sleep behavior, pharmacological therapy—requires further study, as does the evaluation of the impact of this type of intervention on the subsequent disease activity. More research is also required to assess whether sleep impairment might be a prognostic factor for disease flare-ups and for a better understanding of the immunological background between sleep impairment and IBD activity.

## Figures and Tables

**Table 1:** Descriptive statistics for the sociodemographic characteristics in the study and control group.

Characteristics	Study Group		Control Group *n* = 66	Statistic Test	*p*-Value
	Ulcerative Colitis *n* = 76	Crohn’s Disease *n* = 34			
Age, year (mean ± SD)	45.1 ± 14.9		44.3 ± 16.1	1.6957 †	0.4283
	46.2 ± 15.6	42.6 ± 13.5			
Gender (M/F), *n* (%)	36/40	20/14	26/40	3.4368 ‡	0.1793
	(47.4%/52.6%)	(58.8%/41.2%)	(39.4%/60.6%)		
Environment (U/R), *n* (%)	58/18	26/8	42/24	3.28561 ‡	0.1934
	(76.3%/23.7%)	(76.5%/23.5%)	(63.6%/36.4%)		

† Kruskal–Wallis test; ‡ Pearson chi-square test

**Table 2:** Descriptive statistics for biochemical parameters in the study group.

Disease Activity	Study Group		Statistic Test	*p*-Value
	Ulcerative Colitis *n* = 76	Crohn’s Disease *n* = 34		
Active disease, *n* (%)	44 (57.9%)	10 (29.4%)	7.625 ‡	0.0201 *
Mayo or CDAI score, median (Q25; Q75)	3 (1; 7)	129 (43; 200)	-	-
Hemoglobin, (mean ± SD)	12.87 ± 2.33			
	12.55 ± 2.42	13.63 ± 1.95	4.9888 #	0.0276*
Seric iron level, (mean ± SD)	56 ± 34.51	64.25 ± 43.26	1.1026 #	0.2961
Albumin, (mean ± SD)	3.97 ± 0.58	4.05 ± 0.47	0.3582 #	0.5509
Leucocytes, (mean ± SD)	8358 ± 2887	8644 ± 3280	0.2112 #	0.6468
PMN/Ly, median (Q25; Q75)	2.65 (1.72; 3.80)	2.75 (2.26; 3.86)	0.9055 §	0.3651
Platelets, median (Q25; Q75)	314657 (287775; 341540)	325529 (293124; 357934)	0.2267 §	0.6349
ESR 1h, (median (Q25;Q75)	7.8 (5.9;9.8)	6.4 (4.9;7.9)	1.0525 §	0.3083
CRP, (median (Q25;Q75)	2.2 (1.22; 3.19)	3.25 (0.85; 5.64)	0.4269 §	0.6694
CRP/Albumin, (mean ± SD)	0.83 ± 2.32	1.13 ± 2.11	0.3322 #	0.5657
Fibrinogen, median (Q25;Q75)	417 (402; 432)	439 (408; 470)	2.1885 #	0.1424
Fecal calprotectin, median (Q25;Q75)	384.6 (293.9; 475.3)	269.5 (265.2; 272.5)	1.1856 §	0.0237 *
Peripheric arthritis, *n* (%)	14 (18.4%)	2 (5.9%)	3.1473 ‡	0.0345 *
Sacroiliitis, *n* (%)	6 (7.9%)	-	4.5902 ‡	0.0321 *
Pyoderma gangrenosum, *n* (%)	1 (1.3%)	-	-	
Uveitis, *n* (%)	2 (2.6%)	-	-	
Total EIM (% among disease subtype), *n* (%)	21 (27.6%)	2 (5.9%)	17.6101 ‡	0.0001 *
Autoimmune thyroiditis, *n* (%)	6 (7.9%)	2 (5.9%)	0.1463 ‡	0.7072
Arterial hypertension, *n* (%)	16 (21.1%)	4 (11.7%)	0.8094 ‡	0.3683
Diabetes mellitus, *n* (%)	10 (13.2%)	6 (17.6%)	0.3808 ‡	0.5371
Biliary lithiasis, *n* (%)	2 (2.6%)	2 (5.9%)	0.7084 ‡	0.4179

# t-student test; § Mann–Whitney U Test; ‡ Yates, (*) marked effects are significant at *p* < 0.05.

**Table 3:** Descriptive statistics regarding sleep impairment and psychological distress in the study and control group.

Characteristics	Study Group		Control Group	Statistic Test	*p*-Value
	Ulcerative Colitis *n* = 76	Crohn’s Disease *n* = 34			
PSQI, median (Q25;Q75)	8 (5; 10)		4(3; 7)	31.3107 *^*	<0.0001 *
	8 (6; 11)	7(5; 10)			
HADS-A, median (Q25;Q75)	7 (5; 10)		4 (2; 6)	38.605 *^*	<0.0001 *
	8 (5; 10)	7(6; 10)			
HADS-D, median (Q25;Q75)	8 (5; 11)		5 (4; 7)	10.1394 *^*	0.0063 *
	8 (5; 11)	8(4; 10)			

^ Kruskal–Wallis test, (*) marked effects are significant at *p* < 0.05.

**Table 4:** The correlation between sleep quality and disease activity.

PSQI Score vs.	Correlation Coefficient	*p*-Value
Mayo	0.411212	0.0001 *
CDAI	0.0040	0.982

Spearman Rank Order Correlations, (*) marked effects are significant at *p* < 0.05.

**Table 5:** Correlations between PSQI score and clinical and biological parameters.

PSQI Score vs.	Correlation Coefficient	*p*-Value
Age	0.0399	0.6790
Extraintestinal manifestations	0.2754	0.0172 *
Hemoglobin	0.0719	0.460
Fecal Calprotectin	0.4772	0.03608 *
CRP	0.2183	0.0013 *
Fb	0.2073	0.0029 *
PMN/Ly	0.2101	0.0280 *
CRP/Albumin	0.3226	0.0010 *

Spearman Rank Order Correlations, (*) marked effects are significant at *p* < 0.05.

**Table 6:** Correlation between PSQI and Hospital Anxiety and Depression Scale (HADS) score in the inflammatory bowel disease (IBD) group. Univariate analysis.

PSQI Score (PSQI Score ≥5) vs.	Correlation Coefficient	*p*-Value
HADS depression	0.4157	0.00001 *
HADS anxiety	0.4062	0.00001 *
Sleep medication use	0.2062	0.03061 *

Spearman Rank Order Correlations, (*) marked effects are significant at *p* < 0.05.

**Table 7:** Evaluating sleep impairment through each PSQI component in the study and control group.

	Study Group		Control Group (*n* = 66)	Statistic Test	*p*–Value
	RCUH (*n* = 76)	CROHN (*n* = 34)			
PSQI—median (Q25;Q75)	8 (5; 10)	8(6; 10)	4(3; 7)	4(3; 7)*^*	<0.0001 *
Comp 1—subjective sleep quality, *n* (%)	0/1/2/3 12/32/20/12 (13.2%/31.6%/39.5%/15.8%)	0/1/2/3 0/26/6/2 (0%/76.5%/17.7%/5.9%)	0/1/2/3 34/26/4/2 (51.5%/39.4%/6.1%/3.1%)	36.4388 *‡*	<0.0001 *
Comp 2—sleep latency, *n* (%)	12/32/20/12 (15.8%/42.1%/26.3%/15.8%)	10/6/16/2 (29.4%/17.7%/47.1%/5.9%)	18/28/14/6 (27.3%/42.4%/21.2%/9.1%)	15.7472 *‡*	0.0151 *
Comp 3—sleep duration, *n* (%)	8/48/14/6 (10.5%/63.2%/18.4%/7.9%)	6/22/2/4 (17.7%/64.7%/5.9%/11.8%)	26/30/10/0 (39.4%/45.5%/15.2%/0%)	14.2552 *‡*	0.0269 *
Comp 4—habitual sleep efficiency, *n* (%)	20/32/10/14 (26.3%/42.2%/13.2%/18.4%)	14/16/2/2 (41.2%/47.1%/5.9%/5.9%)	52/10/4/0 (78.8%/15.2%/6.1%/0%)	26.0609 *‡*	0.0002 *
Comp 5—sleep disturbances, *n* (%)	4/60/12/0 (5.3%/78.9%/15.8%)	4/28/2/0 (11.8%/82.4%/5.9%)	12/42/12/0 (18.2%/63.6%/18.2%)	9.8583 *‡*	0.0401 *
Wake up for urine emission (yes), *n* (%)	72 (94.7%)	30 (88.2%)	50 (75.8%)	11.1233 *‡*	0.0038 *
Wake up due to diarrhea (yes), *n* (%)	24 (31.6%)	8 (23.5%)	0 (0%)	24.489 *‡*	<0.001 *
Wake up due to nightmares (yes), *n* (%)	26 (34.2%)	12 (35.3%)	18 (27.3%)	1.0184 *‡*	0.5974
Wake up due to pain (yes), *n* (%):	34 (44.7%)	12 (35.3%)	16 (24.2%)	6.5024 *‡*	0.0365 *
Abdominal pain, *n* (%)	16 (21.05%)	6 (17.65%)	2 (3.03%)	-0.5518 *‡*	0.0019 *
Joint pain, *n* (%)	16 (21.05%)	6 (17.65%)	12 (18.18%)	-0.0720 *‡*	0.6579
Headache, *n* (%)	2 (2.63%)	0 (0%)	2 (3.03%)	0.0485 *‡*	0.0915 *
Comp 6—use of sleep medication	66/6/2/2 (86.8%/7.9%/2.6%/2.6%)	28/4/0/2 (82.4%/11.7%/0%/5.9%)	56/6/2/2 (84.9%/9.1%/3%/3%)	2.1993 *‡*	0.9004
Comp 7—daytime dysfunction	12/24/28/12 (15.8%/31.6%/36.8%/15.8%)	0/18/8/8 (0%/52.9%/23.5%/23.5%)	18/30/16/2 (27.3%/45.5%/24.2%/3.1%)	24.1853 *‡*	0.0004 *

^ Kruskal–Wallis test; ‡ Yates or Pearson chi-square test; (*) marked effects are significant at *p* < 0.05.

**Table 8:** The coefficients of multiple linear regression regarding the involvement of each sleep component to the total PSQI score in IBD patient group.

Multiple Linear Regression	Unstandardized Coefficients		Standardized Coefficients	*t*	*p*-Value
	B	Std. Error	Beta		
(Constant)	4.544	0.814		5.579	<0.001 *
Comp 1—subjective sleep quality	3.365	0.264	0.775	12.738	<0.001 *
Comp 5—sleep disturbances	3.312	0.712	0.409	4.653	<0.001 *
Comp 6—use of sleep medication	2.906	0.444	0.533	6.542	<0.001 *
Comp 3—sleep duration	2.884	0.352	0.619	8.192	<0.001 *
Comp 4—habitual sleep efficiency	2.820	0.217	0.781	12.978	<0.001 *
Comp 7—daytime dysfunction	2.508	0.295	0.633	8.505	<0.001 *
Comp 2—sleep latency	2.393	0.286	0.628	8.382	<0.001 *

Model verification: ANOVA: F = 21.648, *p* < 0.001, (*) marked effects are significant at *p* < 0.05.

**Table 9:** The coefficients of multiple linear regression regarding the involvement of each sleep component to the total PSQI score in the control group.

Multiple Linear Regression	Unstandardized Coefficients		Standardized Coefficients	*t*	*p*-Value
	B	Std. Error	Beta		
(Constant)	2.947	0.612		4.816	<0.001 *
Comp 1—subjective sleep quality	2.707	0.340	0.706	7.970	<0.001 *
Comp 4—habitual sleep efficiency	2.534	0.538	0.508	4.712	<0.001 *
Comp 7—daytime dysfunction	2.526	0.310	0.713	8.142	<0.001 *
Comp 5—sleep disturbances	2.083	0.524	0.445	3.976	<0.001 *
Comp 6—use of sleep medication	1.974	0.481	0.456	4.104	<0.001 *
Comp 2—sleep latency	1.958	0.299	0.633	6.548	<0.001 *
Comp 3—sleep duration	1.758	0.456	0.435	3.860	<0.001 *

Model verification: ANOVA: F = 15.806, *p* < 0.001, (*) marked effects are significant at *p* < 0.05.
